# Encapsulation Strategy Matters: Pre- and Post-Loading of Macromolecules into Surface-Supported Microgels Formed via Vaterite Templates

**DOI:** 10.1021/acsmaterialsau.5c00099

**Published:** 2025-10-08

**Authors:** Deniya Joseph, Harrison Brown, Emmanuelle A. B. Konzi, Mehwish Khan, Dmitry Volodkin, Anna Vikulina

**Affiliations:** School of Science and Technology, Department of Chemistry and Forensics, 6122 Nottingham Trent University, Clifton Lane, Nottingham NG11 8NS, United Kingdom

**Keywords:** calcium carbonate, layer-by-layer, adsorption isotherm, hybrid materials, enzymatic degradation

## Abstract

Calcium carbonate vaterite crystals have attracted increasing attention as sacrificial templates for forming polymer microgels. Vaterite’s porous structure, biocompatibility, and eco-friendly synthesis make it ideal for biomedical applications. In this study, vaterite is grown on the surface, and surface-supported (ss)-microgels are formed by coating it with alternating layers of polyelectrolytes, sodium alginate (ALG), and poly-l-lysine (PLL), followed by core dissolution. Pre-loading (during vaterite synthesis) and post-loading (after microgel formation) of macromolecules are compared using dextran and its charged derivatives. Pre-loading proved to be more efficient, achieving up to 9% w/w encapsulation. Dextran adsorption follows the Langmuir model (Δ*G* = – 31.0 kJ/mol), while its derivatives follow the Freundlich model (1/*n* = 0.7–0.8), indicating intermolecular repulsion. Post-loading resulted in encapsulation levels below 1% w/w and exhibited pH-independent behavior. The microgels remained stable in acidic environments, but PLL degradation via trypsin enabled the sustained release of dextran. These findings clarify the mechanisms of macromolecular adsorption on ss-vaterite, highlight the importance of considering the loading strategy when designing microgels for specific applications, and support the use of ss-microgels for therapeutic delivery.

## Graphical abstract

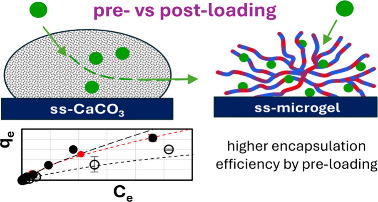

## Introduction

Recent research on polymer microgels for delivery of instructive and therapeutic molecules is gaining significant scientific interest due to the numerous advantages that they provide, including high bioavailability, biodegradation, and mild conditions of synthesis.^1,2^ These benefits are recognized as especially important when encapsulating macromolecules which have a sensitive and fragile nature.^3^

Microgels are cross-linked polymer networks with a 10 nm to 100 μm diameter.^2^ They differ from microcapsules, which consist of a polymer shell surrounding a core (which may be a different material or empty space).^4^ Historically, all microstructures assembled via layer-by-layer (LbL) deposition of polyelectrolytes on colloidal cores were referred to as microcapsules. However, subsequent studies provided a more detailed structural analysis, revealing that many of these are not true capsules but rather two-component gels composed of interpenetrating polymer networks.^5,6^ As a result, they are more accurately described as multilayer microgels.

The process of multilayer microgel fabrication begins with the fabrication of sacrificial templates, one of the most common of which is calcium carbonate microcrystals. Anhydrous CaCO_3_ has three polymorphs: vaterite, calcite, and aragonite.^7,8^ Among them, calcite is the most common and most stable polymorph, which is why it is found in nature. Aragonite is the most difficult CaCO_3_ structure to synthesize, requiring harsher conditions.^9^ The vaterite polymorph is by far the most intriguing CaCO_3_ polymorph, due to its spherical nature and most importantly high porosity, which make vaterite crystals a capable carrier for diverse molecules.^9^ The size of the pores of vaterite crystals is typically in the mesoporous range of a few tens of nanometers, which usually makes the encapsulation of large biomacromolecules more effective when compared to small drugs.^9^ Vaterite can be synthesized under laboratory settings using widely available salts (CaCl_2_ and Na_2_CO_3_) under mild conditions (neutral pH, room temperature).^10^ Compared to other delivery systems like liposomes and dendrimers, these mild synthesis conditions represent a significant advantage, driving extensive research in this area.^9^ The vaterite core can then be sequentially coated with alternating layers of oppositely charged polyelectrolytes, e.g., sodium alginate (ALG) and poly-l-lysine (PLL), and dissolved under mild conditions (e.g., low concentration acid treatment, EDTA), leaving behind a multilayer microgel.^6^

It is noteworthy that recent studies have demonstrated vaterite can be grown directly on surfaces to which it strongly adheres, allowing surface-supported (ss)-microgels to be synthesized.^11^ The entire process of ss-microgel formation is shown in Figure 1. Such ss-microgels provide an alternative surface modification approach that is particularly promising for those biomedical and biotechnological applications that require localized sustained drug release and surface topology controlled at the nano- and microscale.^7,12–16^

**Figure 1:**
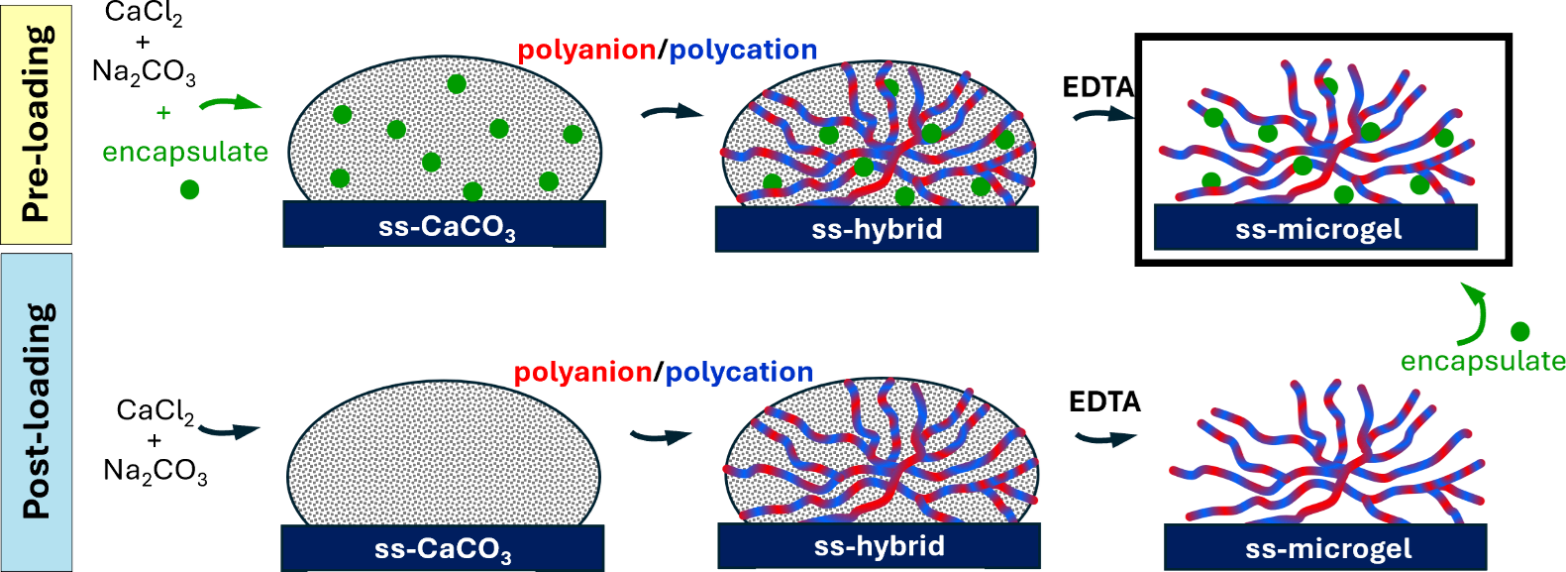
Scheme of in situ growth of ss-CaCO_3_ (gray) on the surface of interest (dark blue), multilayer deposition, and formation of ss-microgels by addition of EDTA, and two approaches for the loading: (upper panel) pre-loading via co-synthesis into the ss-CaCO_3_ crystal; (bottom panel) post-loading onto formed ss-microgels. In this study, ALG has been used as polyanion (red), and PLL as polycation (blue). Dextran^FITC^ and its derivatives (green) were encapsulated into ALG/PLL microgels. The final ss-microgel structure is framed.

There are two main strategies to load microgels with encapsulants (Figure 1).^17^ The first is pre-loading (co-synthesis), where the payload is introduced during the formation of the vaterite crystals. This enables the payload to be trapped inside the crystal pores and remain encapsulated within the microgel after crystal elimination.^17–19^ The second method is post-loading, or adsorption, which involves introducing the payload already after microgel formation so it can bind to the positively and negatively charged polyelectrolytes within the multilayers.^20–22^ Nowadays, the mechanism of loading and the influence of polyelectrolyte–payload interactions on microgel encapsulation capacity and stability remain largely unexplored. There is also insufficient knowledge about optimizing encapsulation efficiency, especially comparing pre-loading (co-synthesis) versus post-loading methods across different types of macromolecules. Besides, nothing is known about the encapsulation into ss-microgels, which was proposed quite recently.

This study compared pre- and post-loading of macromolecular payloads into ALG/PLL ss-microgels templated onto ss-vaterite crystals. Dextran labeled with fluorescein isothiocyanate (FITC) was used as a model neutral encapsulant, alongside its charged derivatives, i.e., diethylaminoethyl (DEAE)-dextran^FITC^ and carboxymethyl (CM)-dextran^FITC^ (chemical structures are shown in Figure S1), to investigate how the charge of the macromolecules influences encapsulation. While none of selected macromolecules are the drugs themselves, they find various applications in drug delivery. Dextran is commonly used in drug delivery systems as a biocompatible polymer carrier.^23,24^ DEAE-dextran promotes electrostatic interactions with negatively charged biomolecules, such as nucleic acids and certain proteins, and facilitates cellular uptake.^25^ CM-dextran has been used to encapsulate cationic molecules through ion-binding processes and amine or ester conjugation, enhancing the stability and controlled release of the drugs.^26^

This study aimed to investigate the mechanism of macromolecule loading into ss-vaterite and reveal how the loading strategy and the charge of macromolecular payloads affect encapsulation efficiency in multilayer ss-microgels, providing insights into their design for controlled delivery of therapeutic macromolecules.

## Experimental Section

### 2.1 Chemicals

Calcium chloride dihydrate (Acros Organics, UK, 10158280), sodium carbonate (Acros Organics, UK, 10577182), ethylenediamine­tetraacetic acid (EDTA) disodium salt dihydrate (Fischer Scientific, UK, 10335460), trypsin-EDTA (Fisher Scientific, UK, 15400054), alginic acid (ALG) sodium salt (Sigma- Aldrich, UK, A-0682), and poly-l-lysine hydrobromide (PLL, 15–30 kDa, Sigma, UK, P7890) were used. Fluorescein isothiocyanate (FITC)-labeled dextrans (various molecular weights), diethyl amino ethyl (DEAE)-dextran^FITC^, and carboxymethyl (CM)-dextran^FITC^ were from Sigma-Aldrich, UK. A stock solution of 250 mM TRIS buffer containing 27 mM KCl and 1370 mM NaCl, pH 7.4 (Alfa Aesar, UK, J60764) was denoted as 10× TRIS and was diluted to 6×, 1×, or 0.2× TRIS by a factor of 1.67, 10, or 50, respectively. Sodium hydroxide 1 M (Fischer Scientific, UK, 10528240) and HCl 1 M (Fischer Scientific, UK, 10467640) were used to adjust the pH of buffer solutions and EDTA. The water used in the experiments was prepared via a Millipore Milli-Q purification system and had a resistivity of >18.2 M Ω·cm.

### 2.2 Formation of CaCO_3_ Crystals and Pre-loading via Co-synthesis

A 0.02 mL amount of 150 mM CaCl_2_, 0.02 mL of 150 mM TRIS (containing 16 mM KCl, 820 mM NaCl), pH 7.4 (TRIS 6×), and 0.02 mL of water or dextran^FITC^, DEAE-dextran^FITC^, or CM-dextran^FITC^ (of desired molecular weight and concentration) dissolved in water, were added into a well in a 96-well plate. These solutions were shaken at 700 rpm for 30 s using an Eppendorf Thermomixer C shaker. A 0.06 mL amount of 50 mM Na_2_CO_3_ was added, and the mixture was agitated by further shaking for 30 s. After shaking, the crystals were left undisturbed to grow for 30 min. ss-CaCO_3_ crystals grew on the surface of a polystyrene 96-well plate. The crystals were visualized using an EVOS Life Technologies imaging system in optical transmittance and fluorescence modes after washing with 0.2× TRIS, pH 8.9. The samples were prepared in duplicates.

### 2.3 Formation of ss-Microgels

The ss-CaCO_3_ crystals were washed twice with 50 μL of 0.2× TRIS buffer with pH adjusted to 8.9 (containing 5 mM TRIS, 0.5 mM KCl, 27 mM NaCl), further denoted as 0.2× TRIS. Following the removal of the washing solution, 50 μL of 0.2× TRIS buffer and 50 μL of 0.5 mg/mL ALG in 0.2× TRIS buffer were added to the well. The well plate was then shaken for 14 min at 700 rpm. Then the solution was removed, and the crystals were washed with 150 μL of 0.2× TRIS by shaking at 700 rpm for 5 min. The washing solution was removed, and 50 μL of 0.2× TRIS buffer and 50 μL of 0.5 mg/mL PLL in 0.2× TRIS buffer were added and shaken at 700 rpm for 14 min. These steps were repeated until five layers, i.e., (polyanion/polycation)_2_-polyanion, were deposited. ss-Microgels were formed by adding 50 μL of 0.2× TRIS buffer along with dropwise addition of 100 μL of 50 mM EDTA pH 7.0. After the coating and ss-microgel formation, the features were visualized using an EVOS Life Technologies imaging system.

### 2.4 Post-loading into ss-Microgels

ss-Microgels were synthesized following the protocol described above using bare ss-vaterite, i.e., prepared without dextran^FITC^ or its derivatives. After the addition of 100 μL of 50 mM EDTA, which resulted in the formation of ss-microgels, the supernatant was replaced with 50 μL of 0.2× TRIS buffer (pH 6.9, 7.9, or 8.9). A 100 μL portion of 150 kDa dextran^FITC^ or its derivatives dissolved in 0.2× TRIS buffer (with pH adjusted to 6.9, 7.9, or 8.9, respectively) was added into a well with ss-microgels and incubated in the dark for 30 min. Simultaneously 50 μL of 0.2× TRIS buffer and 100 μL of 150 kDa dextran ^FITC^ and its derivatives dissolved in 0.2× TRIS buffer were added into empty well plates and incubated in the dark, which served as a control. After the incubation, 100 μL of supernatant was checked for fluorescence using a microplate reader. The samples were prepared in duplicates.

### 2.5 Fluorescence Measurements

Encapsulation efficiency and adsorption capacity of ss-microgels were calculated by measuring residual FITC fluorescence in the supernatant of the crystals after co-synthesis or in the supernatant of ss-microgels using a Thermo Scientific FLUOROSKAN FL microplate fluorometer in black well plates.

### 2.6 Data Analysis

Microscopic images were analyzed using ImageJ software. Average sizes were determined by computing the diameters of 20 particles in their vaterite, multilayer-coated vaterite, and microgel phase. The shrinkage coefficient (%) was determined as a percent ratio of the diameter of microgel to the diameter of the uncoated vaterite crystal measured for at least five particles in the crystal and microgel phase. Percentage yield of vaterite was estimated by counting the number of vaterite and calcite crystals, with the assumption that vaterite crystals have a spherical shape and calcite crystals are cubic. Yield of vaterite was calculated for at least two batches of the crystals fabricated under the same conditions. All data were reported as the mean ± standard deviation (s.d.).

### 2.7 Fitting of Adsorption Isotherms

The adsorption of FITC-labeled dextran and its positively and negatively charged derivatives by mesoporous vaterite crystals was fitted to the Langmuir and Freundlich adsorption models. Equilibrium adsorption capacity was calculated using eq 1:

1 $$q_{e}=\frac{\left(C_{0}-C_{e}\right)\cdot V}{w}$$

where *q*_e_ is the equilibrium adsorption capacity (mg/g), *C*_0_ and *C*_e_ are the initial and equilibrium dextran concentrations, respectively (mg/mL), *V* is the volume of the analyte solution (mL), and *w* is the mass of CaCO_3_ (g), which has been calculated with the assumption of 100% mass yield of calcium carbonate.

The Langmuir and Freundlich models were used for the fitting of pre- and post-loading adsorption isotherms according to eqs 2 and 3, respectively:

2 $$q_{e}=\frac{q_{m}K_{L}}{1+K_{L}C_{e}}C_{e}$$

3 $$q_{e}=K_{F}C_{e}^{1/n}$$

where *K*_L_ is the adsorption equilibrium constant (mL/mg), *K*_F_ is the Freundlich constant (indicates the adsorption capacity), and *n* is the constant of nonlinearity.

### 2.8 Stability of ss-Microgels in HCl

The ss-microgels synthesized by co-synthesis of CM and DEAE derivatives of 150 kDa dextran^FITC^ were examined for stability by incubating them in 0.1 mL of 0.1 M HCl for 1 day and visualization under a microscope for any dissolution, detachment, or morphological changes of ss-microgels prepared in duplicates.

### 2.9 Trypsin-Mediated Degradation of ss-Microgels

ALG/PLL ss-microgels loaded with 500 kDa dextran^FITC^ by co-synthesis and formed onto 96-well plate were degraded by addition of 100 μL of 0.25% Trypsin-EDTA (diluted 10-fold with TRIS buffer, pH 7.3) at room temperature. The images of the same position and fluorescence conditions were taken at different time intervals. Fluorescence profiles were performed to estimate the reduction in fluorescence during the biodegradation. The samples were prepared in duplicates.

## Results and Discussion

### 3.1 Formulation of ss-CaCO_3_ and (ALG/PLL)_2_-ALG ss-Microgels

When encapsulating FITC-labeled dextrans and their derivatives by pre-loading, i.e., co-synthesis with ss-CaCO_3_, they are added to the solution in the first step of crystal formation and therefore can affect the mechanism of crystallization, which has been investigated in this study. ss-CaCO_3_ and (ALG/PLL)_2_-ALG ss-microgels were fabricated according to the protocol described by M. Mammen et al.^11^ using dextran^FITC^ and its derivatives as encapsulants. The vaterite polymorph was a main product in a reaction of co-synthesis with dextran^FITC^ (Figure 2a). ss-Microgels were formed by sequential polymer deposition followed by elimination of the vaterite core (Figure 2c), and the encapsulation of dextran^FITC^ by co-synthesis is confirmed by means of fluorescence microscopy, as illustrated in Figure 2b,d for 500 kDa dextran^FITC^ and is demonstrated in Figures S2 and S3 for 150 kDa dextran^FITC^ and 40 kDa CM-dextran^FITC^, wherein the intermediate step of hybrid (ALG/PLL)_2_-ALG-coated ss-CaCO_3_/dextran^FITC^ crystals is shown. Interestingly, some microgels, e.g., seen in Figures S2 and S3, possess a core–shell structure, while in other cases (Figure 2) they are entirely filled with encapsulants. While some of the shells observed by means of fluorescence microscopy might be due to optical effects, it can also be the case that the microgels have a different internal architecture. Further in this study, only the cumulative amount of encapsulants has been quantified. The distribution of encapsulated molecules within the polymer network of microgels will be considered in future research.

**Figure 2:**
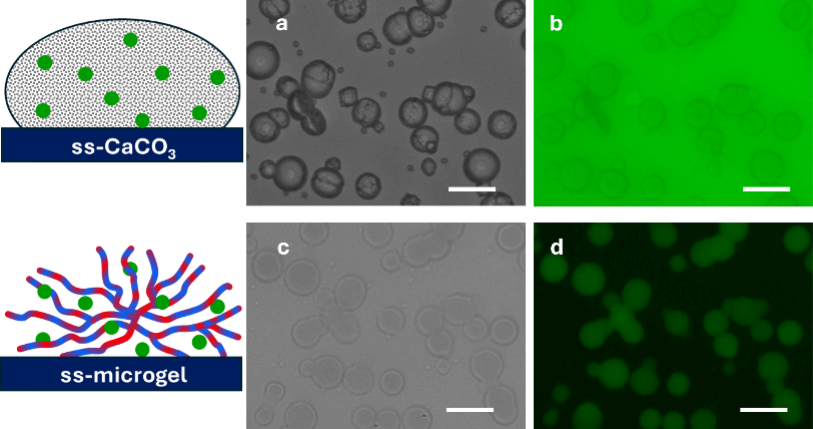
Optical transmittance and fluorescence images of (a, b) ss-CaCO_3_/dextran^FITC^ crystals (before washing out the solution of dextran^FITC^) and (c, d) (ALG/PLL)_2.5_ ss-microgels formed by addition of EDTA. Scale bar is 40 μm. Concentration of 500 kDa dextran^FITC^ is 0.83 g/L. Schematic images on the left side illustrate the stages of fabrication.

The crystals co-synthesized with dextran^FITC^ derivatives which carry negative (CM-) and positive (DEAE-) charge were slightly smaller than bare ss-CaCO_3_, while co-synthesis with dextran^FITC^ did not affect the size of the crystals (Figure 3a). No effect of the concentration of encapsulants in the tested range on the size or morphology of the crystals was found.

**Figure 3:**
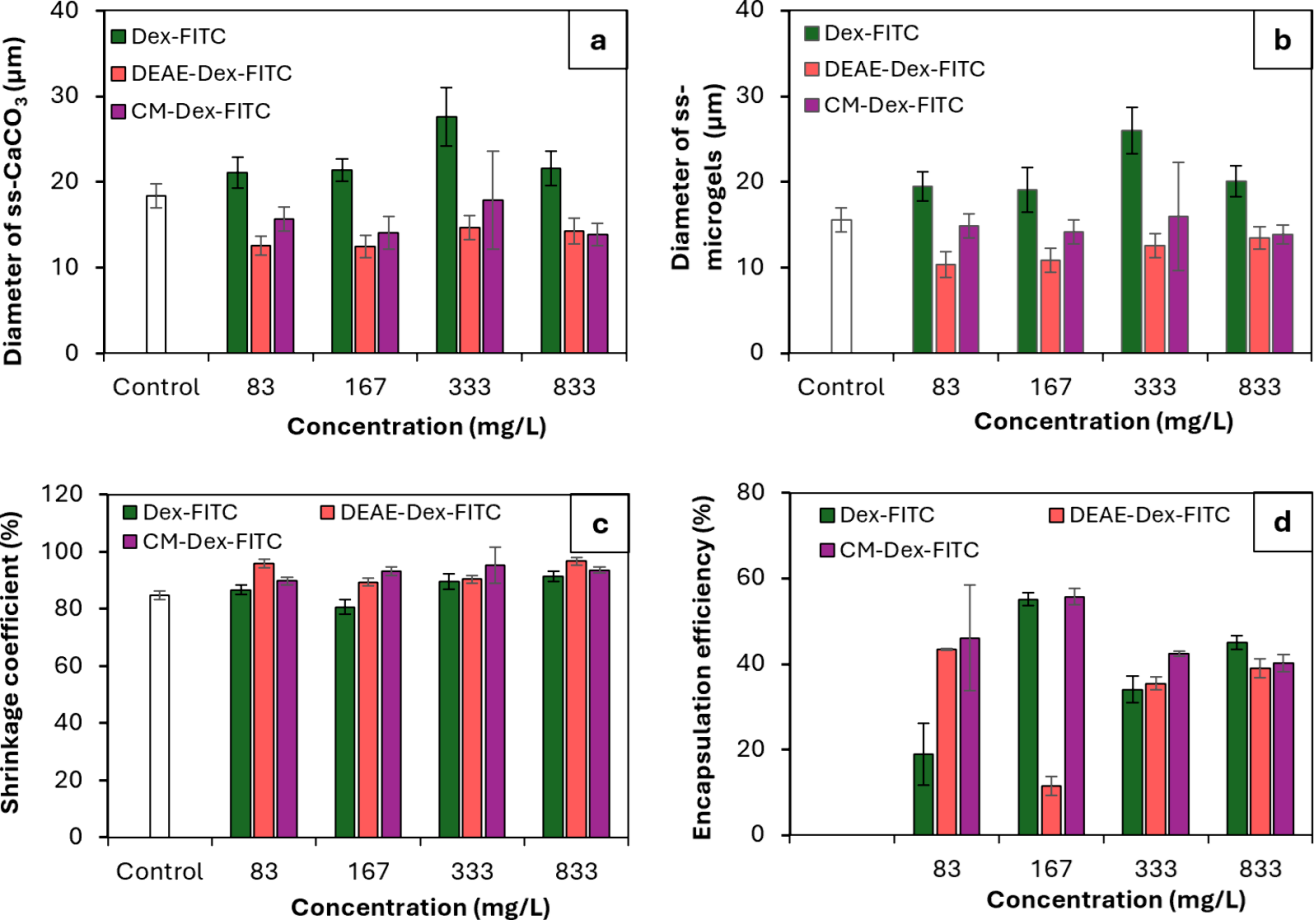
Average diameter of (a) ss-CaCO_3_ crystals and (b) (ALG/PLL)_2.5_ ss-microgels formed by addition of EDTA; (c) shrinkage coefficient of ss-microgels; (d) cumulative encapsulation efficiency of the loading of different dextrans into ss-microgels. 150 kDa dextran^FITC^, DEAE-dextran^FITC^, or CM-dextran^FITC^ has been encapsulated into ss-CaCO_3_ crystals via pre-loading using different concentrations. Control: bare ss-CaCO_3_ crystals and (ALG/PLL)_2.5_ ss-microgels in the absence of any encapsulants.

These results were found to be in good agreement with previous reports for free-standing CaCO_3_,^27^ which indicates that the mechanism of crystal growth on the surface is similar to that in a supersaturated solution. Upon formation, ss-microgels slightly shrank; however, the degree of shrinkage did not depend on the nature and concentration of encapsulated macromolecules (Figure 3b,c).

Regardless of the nature of encapsulated FITC-labeled dextrans, vaterite remained the predominant polymorph, while the fraction of calcite crystals increased in the order CM-dextran^FITC^ ≈ dextran^FITC^ < DEAE-dextran^FITC^.

### 3.2 Quantification of FITC-Labeled Dextran and Its Derivatives

The fluorescence in the FITC-labeled dextran and its charged derivatives was measured to quantify the amount of dextrans encapsulated in the CaCO_3_ crystals. First, the effect of individual Ca^2+^ and CO_3_^2–^ ions as well as their mixture in concentrations equivalent to those in the supernatant of the crystals (*K*_sp_ of 1.4 × 10^–8^ M)^28^ did not influence the fluorescence of dextran^FITC^, DEAE-dextran^FITC^, and CM-dextran^FITC^ (Figure S4).

Similarly, ALG had no pronounced effect on the fluorescence of dextran^FITC^, DEAE-dextran^FITC^, and CM-dextran^FITC^ (Figure S5). PLL suppressed the fluorescence of dextran^FITC^ and CM-dextran^FITC^, which depended on the ratio of PLL:FITC-labeled molecules (Figure S6) and was accompanied by a slight red shift of emission maxima, as shown in Figure S7 for CM-dextran^FITC^, indicating the electrostatic binding of dextran^FITC^ and CM-dextran^FITC^ to the PLL backbone. However, if calculating the amount of dextran^FITC^ and CM-dextran^FITC^ by measuring residual fluorescence in the supernatant, as it has been done in this study, the effect of PLL on fluorescence can be neglected. Calibration curves for the quantification of FITC-labeled encapsulants were constructed by collecting the supernatant of the crystals (for pre-loading) and supernatant of the ss-microgels (for quantification of the encapsulants liberated and lost during ss-microgel formation) and using these supernatants as solvents for calibration standards (Table S1). It is of note that EDTA does not have a pronounced effect on fluorescence of FITC-labeled dextran and its charged derivatives. Interestingly, the retention of dextran-FITC inside the final ss-microgel structure was independent of the number of polyelectrolyte layers, (ALG/PLL)_*n*_, where the tested number of layers ranged from 2 to 6 (data are not shown).

### 3.3 Pre- vs Post-loading Approaches

Co-synthesis and adsorption approaches for the encapsulation of 150 kDa FITC-labeled dextran and its CM and DEAE derivatives have been compared by constructing adsorption isotherms in the same concentration range (Figure 4).

**Figure 4:**
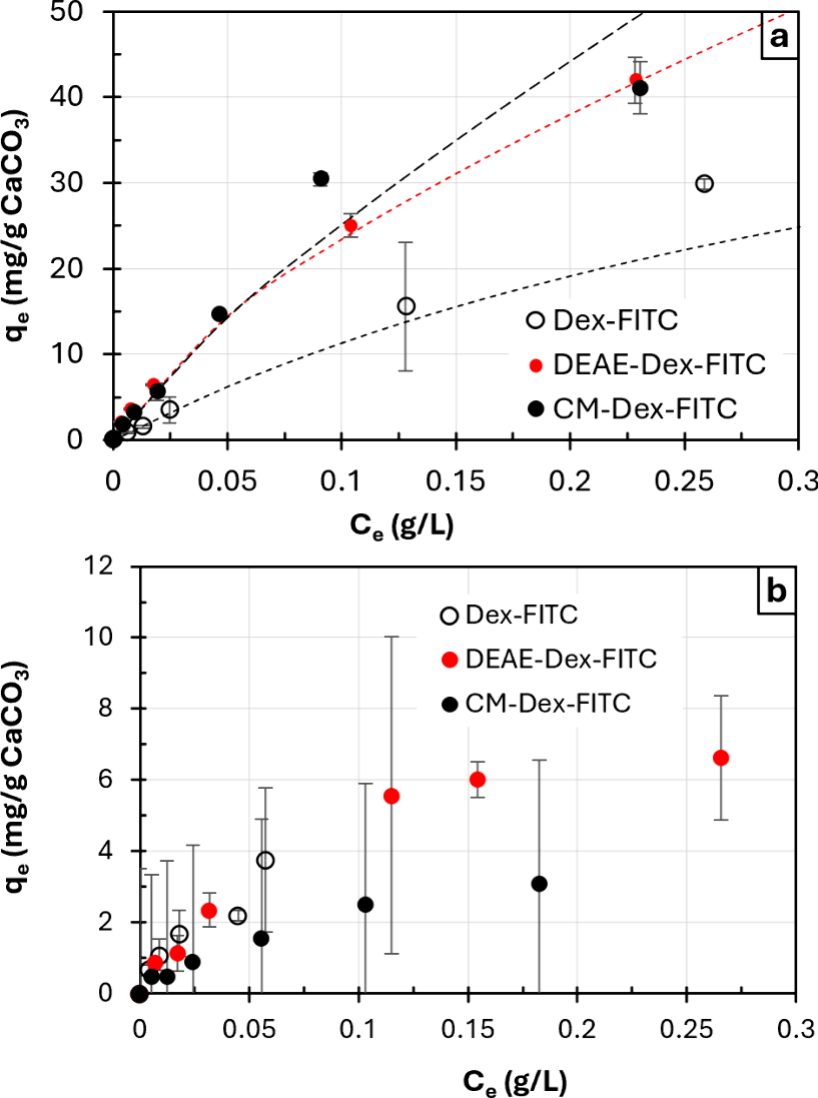
Isotherms of adsorption of 150 kDa dextran^FITC^, DEAE-dextran^FITC^, or CM-dextran^FITC^: (a) into ss-CaCO_3_ via co-synthesis; (b) into (ALG/PLL)_2.5_ ss-microgels via post-loading. The values are the mean ± s.d. (*n* = 2).

It is of note that the quantification of post-loading was barely possible, as the decrease in supernatant fluorescence was close to the limit of quantification, which also resulted in higher error bars in Figure 4b. Nevertheless, it is clearly seen that, regardless of the nature of the encapsulants, the adsorption capacity of ss-microgels was found to be approximately an order of magnitude higher for co-synthesis of the crystals. Aiming to improve the post-loading of dextran^FITC^ and its CM and DEAE derivatives, the pH of the solution has been changed, with the thought to shift the charge balance and increase the electrostatic attraction of encapsulants to ss-microgels. However, the variation in pH in the range from 6.9 to 8.9 did not affect the adsorption (Figure S9). These results suggest that co-synthesis is a more effective way for the encapsulation of macromolecular cargos into multilayer microgels.

In order to reveal a physical-chemical mechanism of adsorption onto ss-vaterite, the adsorption isotherms shown in Figure 4a have been fitted to Langmuir and Freundlich models. The linearization was done using Lineweaver–Burk and logarithmic coordinates for Langmuir and Freundlich models, respectively (Figure S10). Table 1 summarizes the parameters and coefficients of determination calculated for the two model. The Langmuir model, which describes physisorption, was a better fit for dextran^FITC^. The adsorption is driven by a free Gibbs energy Δ*G* of −31.0 kJ/mol. The maximum adsorption capacity was found to be 61.7 mg/g CaCO_3_, which is equivalent to ∼90 mg/g microgels, or 9% _w/w_ if assuming no shrinkage, and the densities of ∼2.6 and ∼1.1 g/cm^3^ for CaCO_3_ ^29^ and the polyion in a multilayer capsule,^30^ respectively. In contrast, adsorption of DEAE- and CM-dextran^FITC^ followed the Freundlich model with 1/*n* < 1. This indicates the adsorption of charged dextran^FITC^ derivatives onto the heterogeneous surface. In the application to ss-vaterite, this might be explained by electrostatic repulsion between adsorbate molecules, which resulted in the decay in the energy distribution of adsorbed sites.

**Table 1:** Summary of the Fitting Parameters for the Isotherms of Adsorption of 150 kDa dextran^FITC^, DEAE-dextran^FITC^, or CM-dextran^FITC^ into ss-CaCO_3_ via Co-synthesis to the Langmuir and Freundlich Models

	Langmuir parameters			Freundlich parameters			
Adsorbate	*Q*_max_, mg/g CaCO_3_	*K*_L_, L/g	*R* ^2^	1/*n*	*K*_F_	*R* ^2^	Adsorption model
dextran^FITC^	61.7 ± 11.8	2.3 ± 0.4	0.9920	1.08 ± 0.08	184 ± 117	0.9767	Langmuir
DEAE-dextran^FITC^	19.1 ± 9.9	40 ± 22	0.9524	0.70 ± 0.03	117 ± 23	0.9973	Freundlich
CM-dextran^FITC^	27.6 ± 16.3	18 ± 11	0.9755	0.81 ± 0.07	164 ± 81	0.9810	

It is interesting to note that, according to previous reports, adsorption of globular macromolecules (proteins) onto vaterite follows the Langmuir model.^10,18^ Apparently, polysaccharide chains containing charged groups are prone to higher intermolecular electrostatic interactions compared to the compact structure of globular proteins, which changes the mechanism of adsorption from Langmuir physisorption to Freundlich chemisorption.

In addition to the Langmuir and Freundlich models, fitting to Temkin and Dubinin–Radushkevich isotherms of adsorption was performed (Figure S11) to consider the models that might be appropriate for the adsorption onto the porous surfaces. While the Temkin isotherm is not specifically designed for mesoporous materials, it describes adsorption where the heat of adsorption decreases linearly with surface coverage and therefore might be relevant when assessing chemical adsorption. The Dubinin–Radushkevich model is primarily used to analyze adsorption onto microporous materials, describing the pore-filling adsorption mechanism. The goodness of the fit was poor for the Temkin isotherm, with *R* ^2^ values below 0.9 for all three encapsulants. Better *R* ^2^ values of 0.96 were obtained for the Dubinin–Radushkevich model, especially for DEAE- and CM-dextran^FITC^, although the Freundlich model still provides the best fitting from all four tested models. These results indicate that the pore-filling mechanism also might play a role in dextran-FITC and its derivatives’ adsorption.

### 3.4 ss-Microgel Degradation and Mediated Release of Encapsulants

The ss-microgels fabricated by co-synthesis of CM and DEAE derivatives of 150 kDa dextran^FITC^ were examined for chemical stability by incubating them in 0.1 M HCl for 1 day and visualization under a microscope for any dissolution or morphological changes of the microgels. The microgels are prone to some deformation and shrinkage (reduced in size to 22 ± 8%) when exposed to 0.1 M HCl; however, they do not detach from the surface and therefore possess high stability in an acidic environment (Figure 5). This might open avenues for post-loading of the encapsulants into ss-microgels from acidic solutions, for instance, using the approach described for bovine serum albumin.^31^

**Figure 5:**
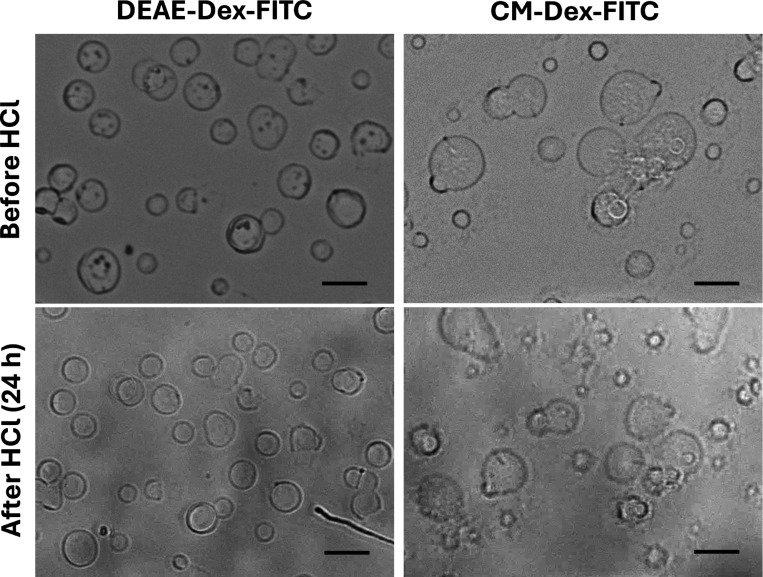
Optical transmittance images of (ALG/PLL)_2.5_ ss-microgels loaded with DEAE- (a, b) or CM-dextran^FITC^ (c, d) via co-synthesis (C_0_ 3.33 g/L) before and after incubation in 0.1 M HCl for 1 day. Brightness/contrast are adjusted. Scale bar: 20 μm.

Degradation of multilayer microgels by cleavage of peptide bonds in PLL using trypsin was demonstrated earlier. However, the kinetics of the release of encapsulated molecules mediated by enzymatic degradation remained unclear. In this study, the degradation-mediated release of dextran^FITC^ has been investigated by means of fluorescence microscopy (Figure 6).

**Figure 6:**
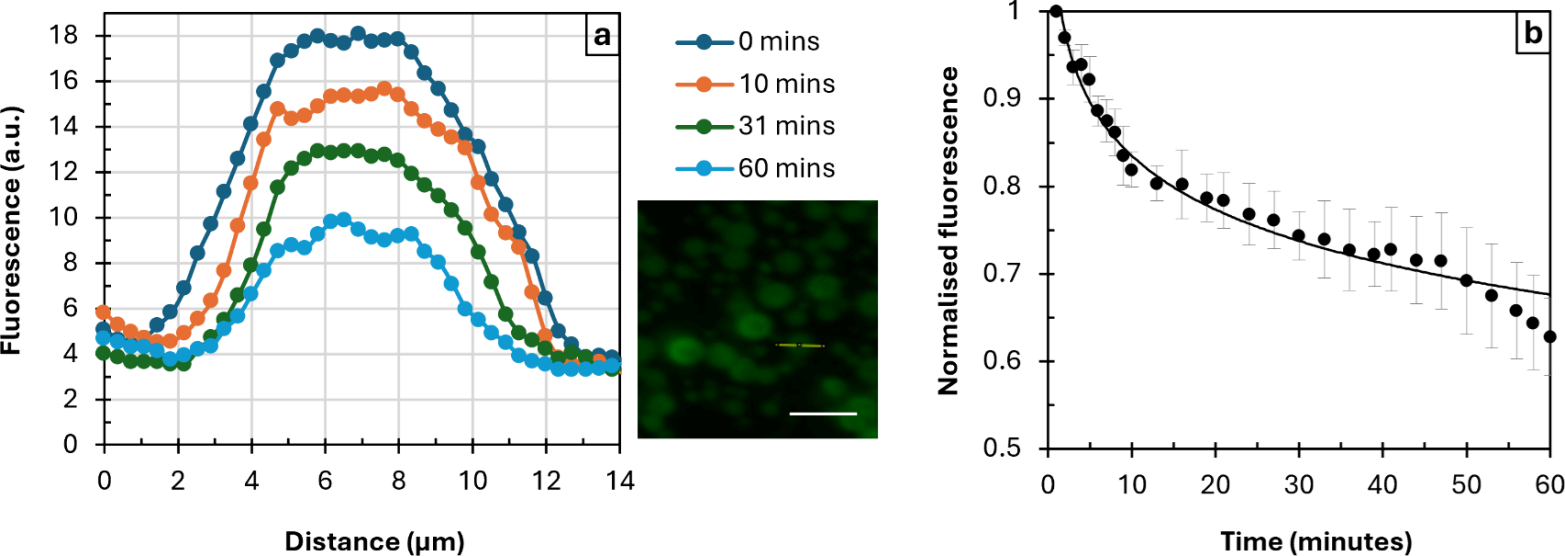
(a) Cross-section profiles of an (ALG/PLL)_2.5_ ss-microgel loaded with 150 kDa dextran^FITC^ at different times of incubation in trypsin-EDTA. Fluorescence image shows the ss-microgel before addition of trypsin-EDTA. Scale bar is 20 μm. (b) Kinetics of degradation-mediated release of dextran^FITC^ from ss-microgels under static conditions. The values are mean ± standard deviation (*n* = 3).

Upon degradation, ss-microgels did not significantly decrease in size but released encapsulated dextran^FITC^ (Figure 6a), which followed an exponential-like decay (Figure 6b). The kinetics has been observed for 60 min; ∼35–40% of dextran^FITC^ has been released from ss-microgels in 1 h under static conditions. It is of note that there is no decay of fluorescence in a control experiment without trypsin (data are not shown).

## Conclusion

The recently developed method for fabricating hard-templated multilayer microgels directly on surfaces represents a transformative advance for modifying material interfaces with a primary application of medical surfaces. This approach provides a robust alternative to classical multilayer coating techniques, enabling the creation of microparticulate, biopolymer-based coatings with enhanced functionality. Our study rigorously compared two encapsulation strategies, i.e., pre-loading via co-synthesis with ss-vaterite CaCO_3_ and post-loading onto preformed ss-microgels, using dextran^FITC^ and its charged derivatives as representative macromolecules. The results demonstrate that pre-loading yields significantly higher encapsulation efficiency. Attempts to optimize post-loading through pH-modulated charge screening proved ineffective within the pH range of 6.9–8.9. Adsorption isotherms aligned with Langmuir and Freundlich models, revealing distinct mechanisms governed by physisorption and electrostatic interactions. Importantly, ALG/PLL ss-microgels exhibit exceptional chemical stability in acidic environments yet undergo enzymatic degradation via trypsin, which mediates the release of encapsulants. These findings establish ss-microgels as a powerful platform for the encapsulation and sustained delivery of therapeutic macromolecules, which may find applications in tissue engineering and medical biocoatings.

## Supplementary Material



## Notes

Figure S1. Structure of FITC-labeled polysaccharides; Figures S2, S3. Optical transmittance and fluorescence images of ss-crystals and microgels; Table S1. Comparison of calibration curve parameters; Figures S4–S7. Effect of ions, ALG, and PLL on the fluorescence of dextran^FITC^ and its derivatives; Figure S8. Fluorescence of supernatant collected after different steps of ss-microgel fabrication; Figures S9–S11 and eqs S1, S2. Adsorption isotherms and their fitting in linear coordinates (PDF)

## ABBREVIATIONS

ALG: sodium alginate
FITC: fluorescein isothiocyanate
LbL: layer-by-layer
PLL: poly-l-lysine
