# Supplementary material for: Encapsulation Strategy Matters: Pre- and Post-Loading of Macromolecules into Surface-Supported Microgels Formed via Vaterite Templates
Source: ACS Mater Au. 2025 Oct 8;5(6):1001–8. doi: 10.1021/acsmaterialsau.5c00099 (PMC12616436; doi:10.1021/acsmaterialsau.5c00099)
Supplement: Supplementary file 1 [file mg5c00099_si_001.pdf]

SUPPORTING INFORMATION

# ENCAPSULATION STRATEGY MATTERS: PRE- AND POST-LOADING OF MACROMOLECULES INTO SURFACE-SUPPORTED MICROGELS FORMED VIA VATERITE TEMPLATES

Deniya Joseph, Harrison Brown, Emmanuelle A. B. Konzi, Mehwish Khan, Dmitry Volodkin, Anna Vikulina*

School of Science and Technology, Department of Chemistry and Forensics, Nottingham Trent University, Clifton Lane, Nottingham NG11 8NS, United Kingdom

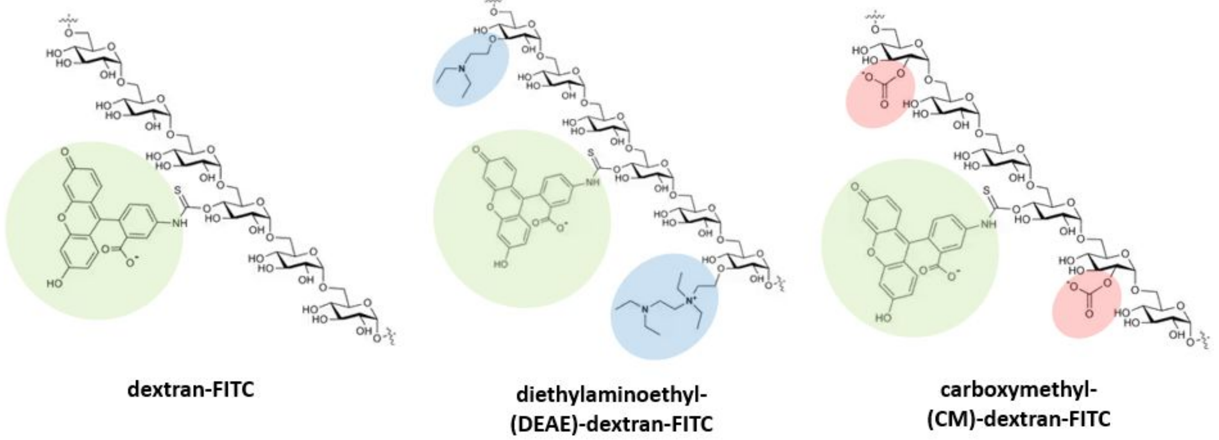


**Figure S1.** Structure of FITC-labelled polysaccharides.

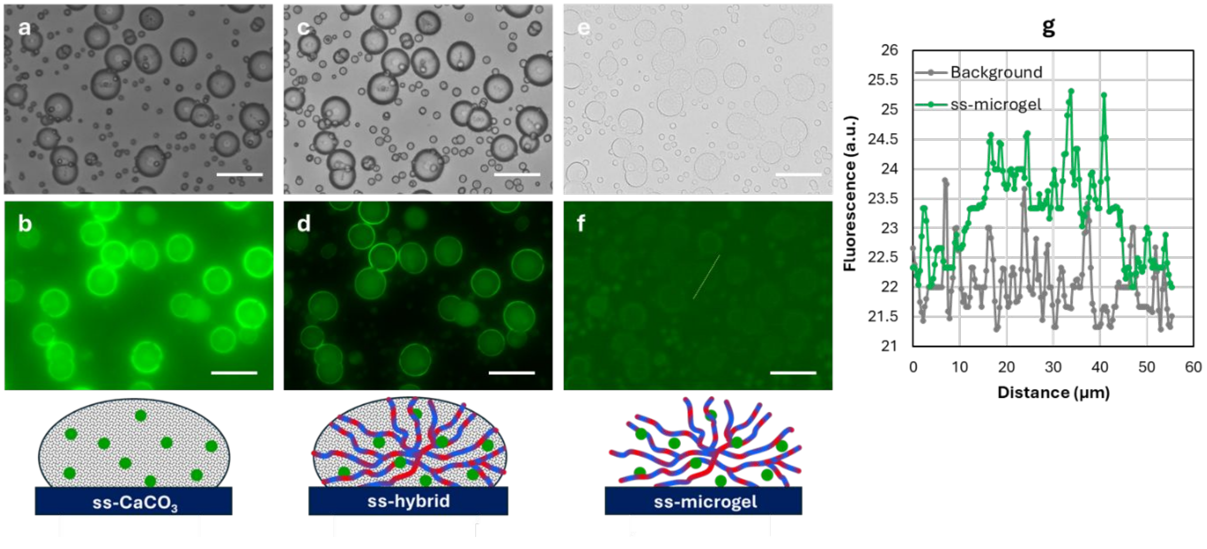


**Figure S2.** Optical transmittance and fluorescence images of: **a,b** - ss-dextran$^{FITC}$/$CaCO_3$ crystals (after washing out the solution of dextran$^{FITC}$), **c,d** - ss-dextran$^{FITC}$/$CaCO_3$-(ALG/PLL)$_{2.5}$ hybrid crystals, **e,f** - (ALG/PLL)$_{2.5}$ ss-microgels formed by addition of EDTA. Scale bar is 40 µm. The encapsulation of 150 kDa dextran$^{FITC}$ via pre-loading (initial concentration of 0.83 mg/mL). g: fluorescence profiles taken across ss-MG (yellow line in image f) and background (not crossing any ss-microgels).

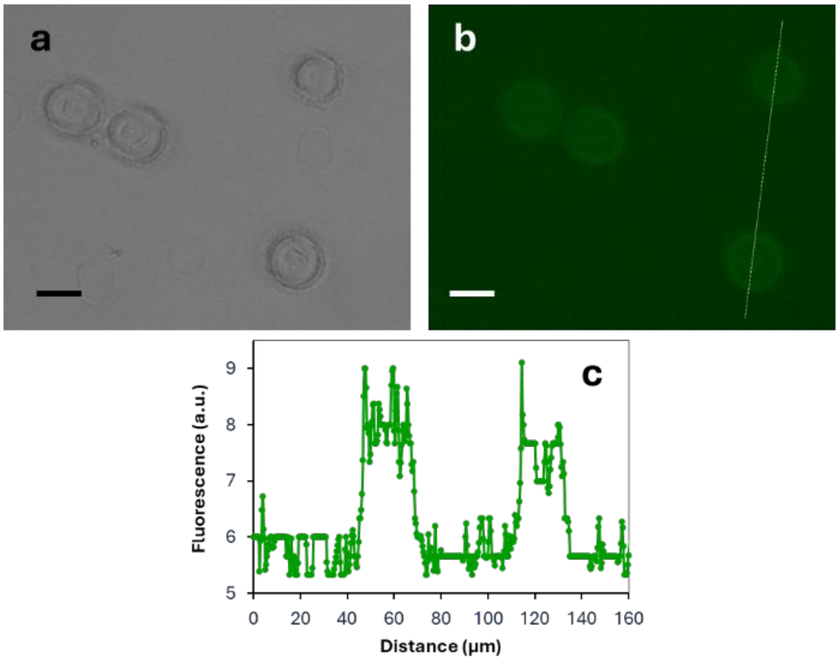


**Figure S3. a,b** - Optical transmittance and fluorescence images of: $(ALG/PLL)_{2.5}$ ss-microgels formed by addition of EDTA. Scale bar is 20 µm. The encapsulation of 40 kDa CM-dextran$^{FITC}$ via pre-loading (initial concentration of 0.83 mg/mL). **c** - Fluorescence profiles taken across two ss-microgels (yellow line in image b).

**Table S1.** Comparison of calibration curve parameters calculated for different FITC-dextrans for different media: EDTA solution used for the elimination of crystal templates was collected after 30 minutes of incubation and used as a solvent for FITC-dextrans. This set of calibration curves was used for post-loading adsorption calculations. The supernatant of bare crystals has been collected after crystal formation and used as a solvent for FITC-dextrans. This set of calibration was used for pre-loading (co-synthesis) adsorption calculations.

| | DEX-FITC | | DEAE-DEX-FITC | | CM-DEX-FITC | |
|---|---|---|---|---|---|---|
| Medium | EDTA collected from microgels | Supernatant of crystals | EDTA collected from microgels | Supernatant of crystals | EDTA collected from microgels | Supernatant of crystals |
| Slope, mL/mg | 3555 ± 163 | 3276 ± 140 | 1041 ± 25 | 1012 ± 32 | 912 ± 64 | 1151 ± 34 |
| Intercept | -38.9 ± 25.0 | 15.0 ± 22.7 | -3.1 ± 3.3 | 2.1 ± 5.3 | -18.0 ± 10.5 | 0.9 ± 5.5 |
| $R^2$ | 0.9826 | 0.9892 | 0.9965 | 0.9938 | 0.9854 | 0.9947 |

**Effect of ions on fluorescence of dextran$^{FITC}$ and its derivatives**

To investigate individual effect of calcium ions, carbonate ions and the presence of both ions on the fluorescence of DEX $^{FITC}$ and its derivatives, fluorescence of 0.2 mg/mL 150 kDa dextran$^{FITC}$ / DEAE-dextran$^{FITC}$ / CM-dextran$^{FITC}$ in 1x TRIS buffer pH 7.4 was measured in the absence and in presence of 0.1 mM calcium chloride, 0.1 mM sodium carbonate, or their mixture. The fluorescence was measured using Microplate Reader from 200 µL of solution, the samples were prepared in duplicates**.**

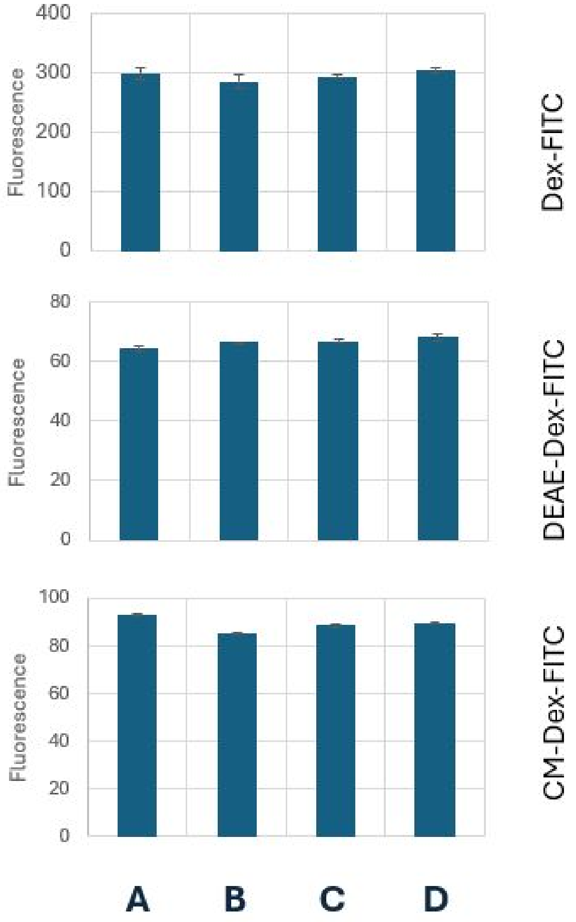


**Figure S4.** Effect of $Ca^{2+}$ and $(CO_3)^{2-}$ ions (0.1 mM) on fluorescence of dextran$^{FITC}$(top), DEAE-dextran$^{FITC}$ (middle) and CM-dextran$^{FITC}$ (bottom). Concentration of dextran$^{FITC}$ and its derivatives is 0.2 mg/mL in A - TRIS buffer, B - TRIS buffer in the presence of 0.1 mM $CaCl_2$, C - TRIS buffer in the presence of 0.1 mM $Na_2CO_3$, D - TRIS buffer in the presence of 0.1 mM $CaCl_2$ and $Na_2CO_3$.

## Effect of PLL and ALG on fluorescence of dextran$^{FITC}$ and its derivatives

The effect of PLL and ALG on fluorescence of 150 kDa FITC-labelled dextran and it's cationic and anionic derivatives was investigated by changing the mass ratio of the dextran to polymer in the range of 10:0 (control, without ALG or PLL), 10:1, 5:1, 2:1, 1:1, and 1:10, at fixed concentration of dextran$^{FITC}$ at 0.2 mg/mL. Fluorescence was measured using Microplate Reader and Nanodrop Fluorospectrometer. The samples were prepared in duplicates.

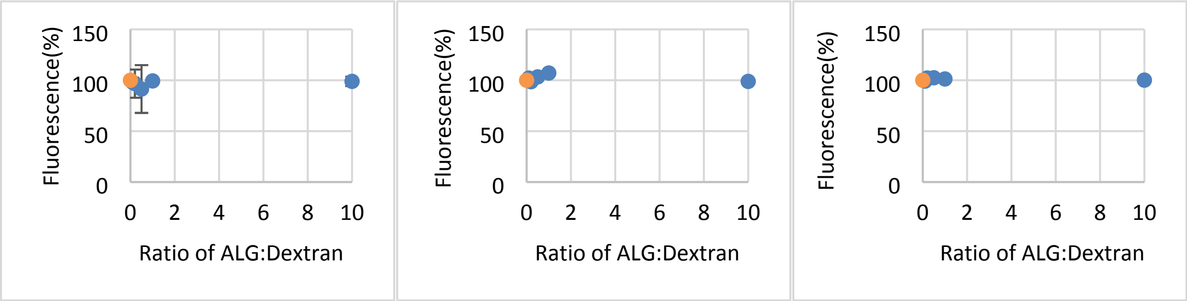


**Figure S5.** Effect of PLL on fluorescence of dextran$^{FITC}$ (left), DEAE-dextran$^{FITC}$ (middle), CM-dextran$^{FITC}$ (right). Fluorescence in the absence of ALG is taken as 100% (orange datapoints).

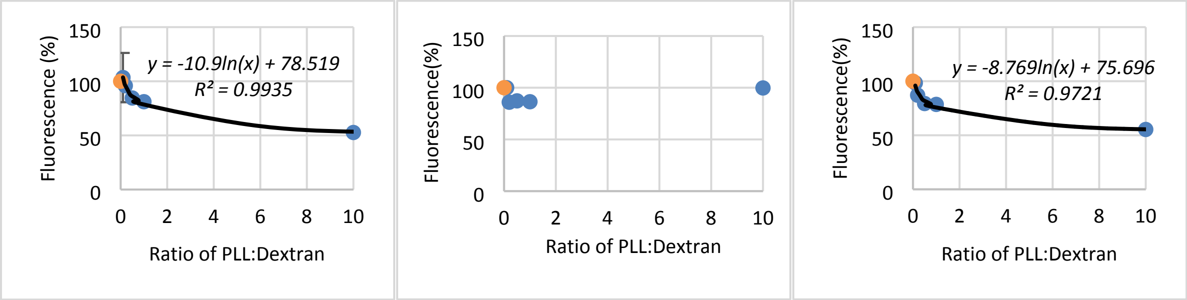


**Figure S6.** Effect of PLL on fluorescence of dextran$^{FITC}$ (left), DEAE-dextran$^{FITC}$ (middle), CM-dextran$^{FITC}$ (right). Fluorescence in the absence of PLL is taken as 100% (orange datapoints).

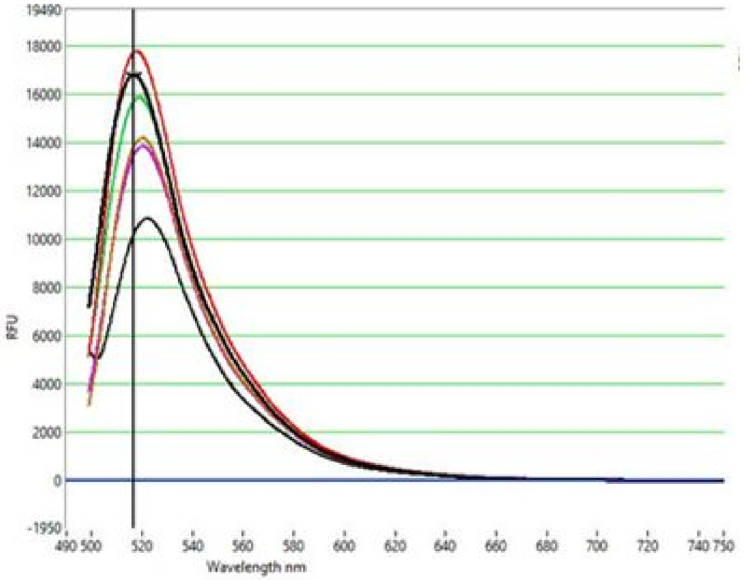


**Figure S7.** Effect of PLL on emission spectrum of CM-dextran$^{FITC}$ in different CM-dextran$^{FITC}$ : PLL mass ratio: 10:0 (red), 10:1 (black bold), 5:1 (green), 2:1 (orange), 1:1 (pink), and 1:10 (black).

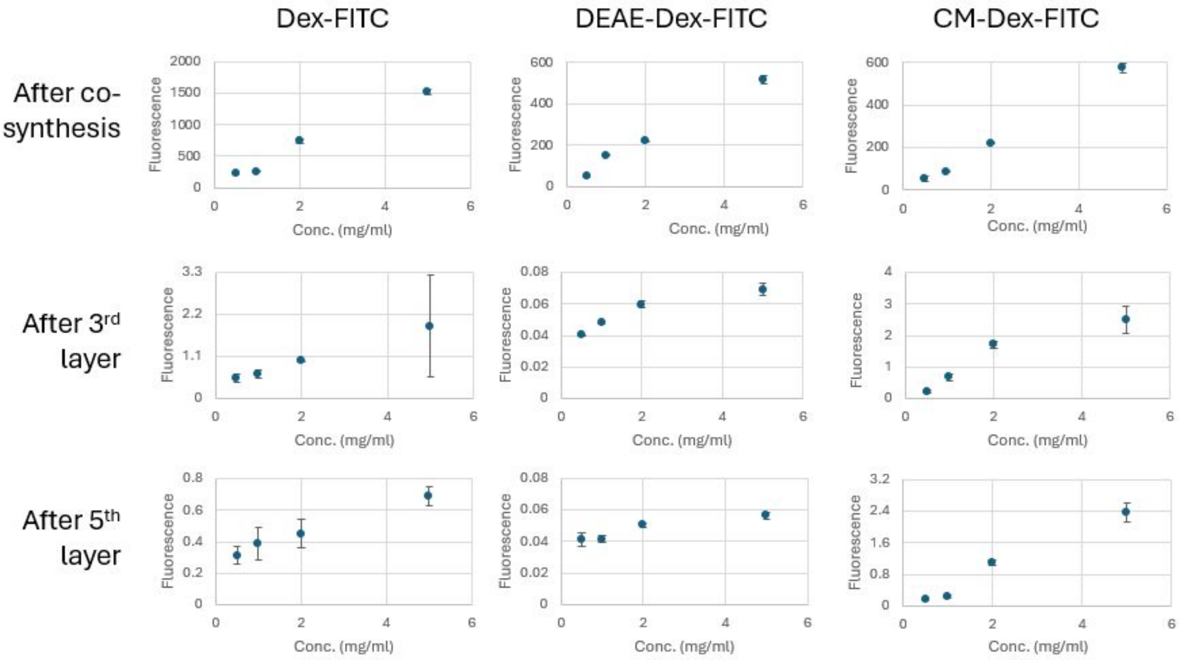


**Figure S8.** Fluorescence (a.u.) of supernatant collected after co-synthesis (upper panel) and after deposition of the 3rd (middle) and 5th polymer layer (bottom panel). 150 kDa dextran$^{FITC}$, DEAE-dextran$^{FITC}$ or CM-dextran$^{FITC}$ have been encapsulated into ss-$CaCO_3$ crystals via pre-loading using different concentrations (stock solution concentrations are given on the graph). The values are mean ± s.d. (n = 2).

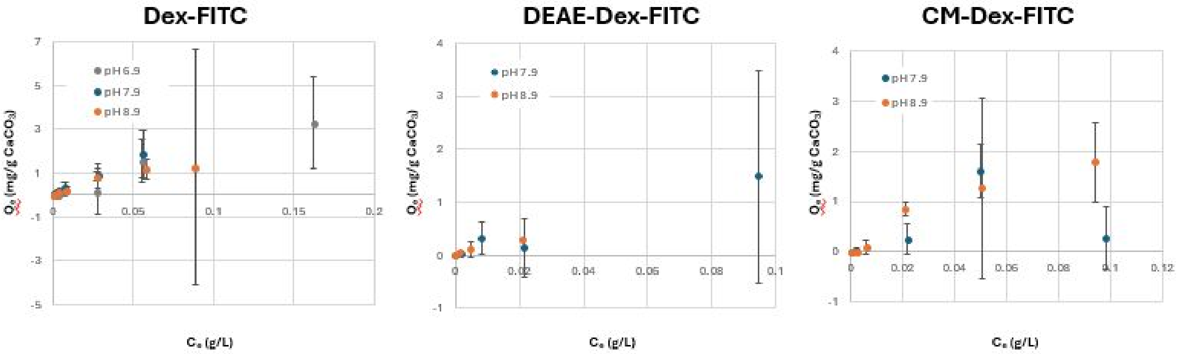


**Figure S9.** Isotherms of adsorption of 150 kDa dextran$^{FITC}$, DEAE-dextran$^{FITC}$ and CM-dextran$^{FITC}$ into $(ALG/PLL)_{2.5}$ ss-microgels via post-loading at different pH. The values are mean ± s.d. (n = 2). For DEAE-dextran$^{FITC}$ and CM-dextran$^{FITC}$, no quantification could be done for pH 6.9 (obtained values were below limit of quantification).

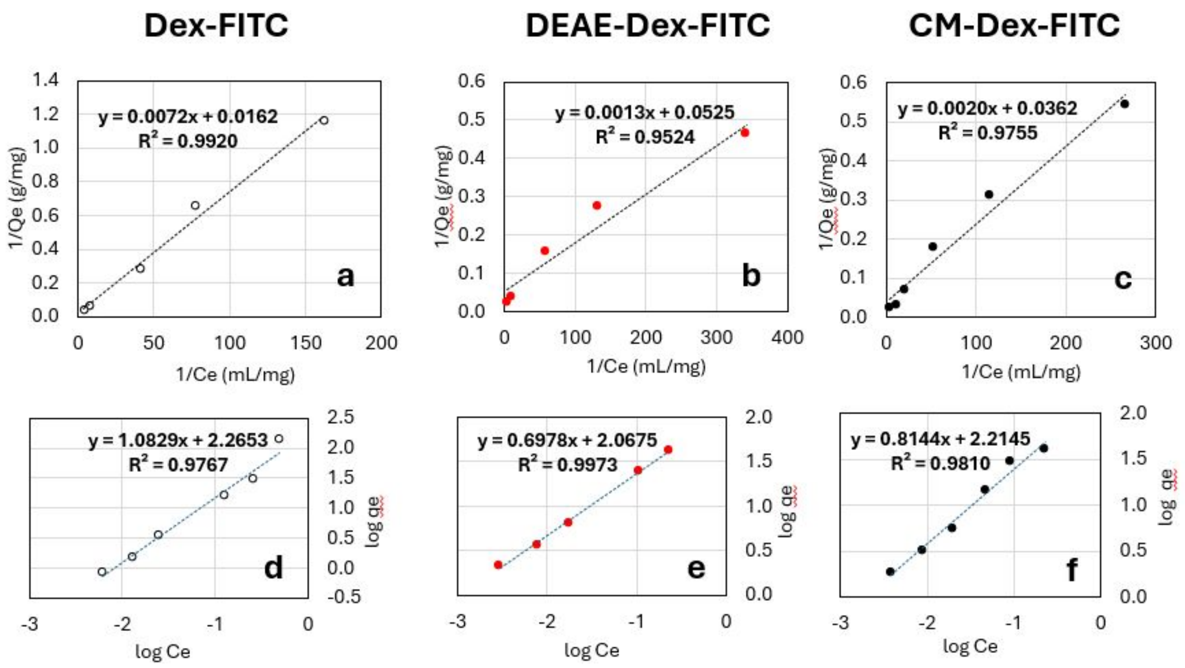


**Figure S10.** Lineweaver-Burk (a-c) and Freundlich (d-f) plots for the isotherms of co-synthesis (pre-loading) of 150 kDa dextran$^{FITC}$, DEAE-dextran$^{FITC}$ and CM-dextran$^{FITC}$.

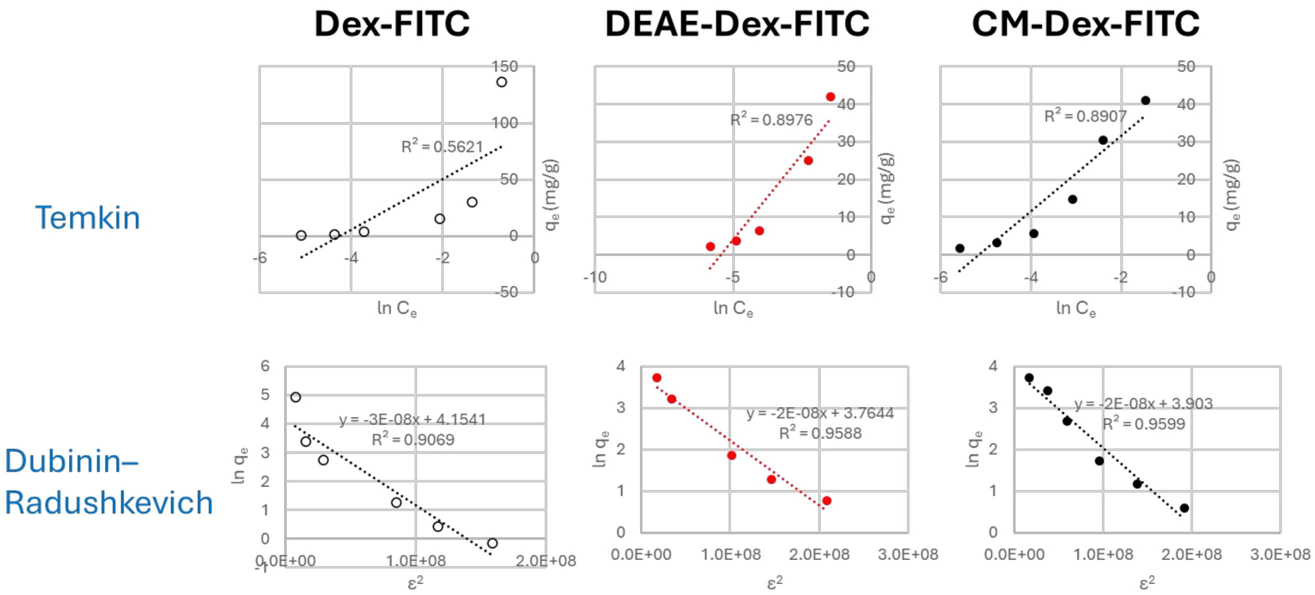


**Figure S11.** Temkin and Dubinin-Radushkevich isotherms in linear coordinates (eq. S1 and S2, respectively) for the co-synthesis (pre-loading) of 150 kDa dextran$^{FITC}$, DEAE-dextran$^{FITC}$ and CM-dextran$^{FITC}$. where, $q_e$ and $q_{max}$ are the equilibrium and maximum adsorption capacity, respectively (mg/g), $c_e$ is the equilibrium dextran concentrations (g/L), $b_t$ and $A_t$ are Temkin isotherm constants, R is the universal gas constant (8.314 J/mol·K), T is the temperature (K).

$$q_e = \frac{RT}{b_t} \ln A_t + \frac{RT}{b_t} \ln c_e \qquad (Eq. S1)$$

$$\ln q_e = \ln q_{max} - \beta \varepsilon^2, where\ \varepsilon = RT \ln(1 + \frac{1}{c_e}) \qquad (Eq. S2)$$
